# The number of acute cerebrovascular events in Israel: a forecast until 2040

**DOI:** 10.1186/s13584-019-0337-1

**Published:** 2019-10-01

**Authors:** Assaf Ben Shoham, Sigal Liberant-Taub, Mor Sharon, Inbar Zucker

**Affiliations:** 1 Smokler center for health policy research, Myers-JDC-Brookdale Institute, P.O.B. 3886, 91037 Jerusalem, Israel; 2 Family Medicine, Clalit health services, Jerusalem, Israel; 3 General medicine, Ministry of Health, Jerusalem, Israel; 4 Israeli Center for Disease Control (ICDC), Ministry of Health, Jerusalem, Israel; 5 School of Public Health, Sackler Faculty of Medicine, Tel Aviv University, Tel Aviv, Israel

**Keywords:** Acute cerebrovascular event, Forecast, Epidemiology, Aging, Ethnicity

## Abstract

**Background:**

Acute cerebrovascular event is one of the leading causes of death in Israel and is the primary cause of neurological disability in adults. Although some evidence indicates that the incidence rate of acute cerebrovascular events in developed countries is stable or has been decreasing over the past decades, the number of events is expected to increase in these countries due to projected changes in size and composition of the population. The purpose of this study was to provide a forecast of the number of acute cerebrovascular events in Israel for the coming decades.

**Methods:**

We used data from the National Stroke Registry at the Israel Center for Disease Control and data from the long-term population forecasts of the Israeli Central Bureau of Statistics. We generated forecasts of the annual number of acute cerebrovascular events based on the mean annual incidence rates during 2014–2016 within population subgroups defined by gender, age, and ethnicity, and on the projected population size of these subgroups for 2015–2040. The forecasts were generated for various assumptions as to trends in the incidence rate and for alternatives as to the projected population growth.

**Results:**

Based on the intermediate population growth alternative, the annual number of acute cerebrovascular events is expected to increase from 18,400 to 38,500, 34,800 or 26,400 events, assuming constant annual incidence rates, decreasing annual incidence rates at a rate of 2% every 5 years, or decreasing annual incidence rates at a rate of 7.25% every 5 years, respectively. Whereas, presently, events affecting Arab patients account for 15% of acute cerebrovascular events and events affecting patients over 80 account for 33% of acute cerebrovascular events, by 2040 events affecting Arab patients will account for more than 21% of the events and events affecting patients over 80 will account for 42% of the events.

**Conclusions:**

In view of the expected increase in the number of acute cerebrovascular events and the changes in the demographic composition of adults suffering from such events, and in order to allow for optimal care and equity, it is imperative to evaluate the preparedness of care provision and the geographical deployment of treatment services in the short and long term.

**Electronic supplementary material:**

The online version of this article (10.1186/s13584-019-0337-1) contains supplementary material, which is available to authorized users.

## Background

Acute cerebrovascular event is one of the leading causes of death in Israel, accounting for approximately 5.5% of all deaths, and is the primary cause neurological of disability in adults [1, 2]. Some studies estimate that about a third of event survivors are disabled, have poor post-event cognitive ability and poor mental health [3]. Acute cerebrovascular events are associated with an increased economic burden, incorporating in-hospital acute-care costs, and continuation, rehabilitation and long-term costs in the community. In addition to direct health care costs, cerebrovascular events involve societal costs including informal care and productivity loss due to death and disability. Cerebrovascular events are the second most common cause of global disability-adjusted life-years (DALYs), a measure of overall disease burden. In Israel, cerebrovascular events due to ischemic stroke and hemorrhagic stroke during 2016, were estimated to account for more than 45,000 DALYs [4].

Recently, the Medical Directorate in the Ministry of Health (MOH) launched a National Plan for the Treatment and Prevention of Stroke Damage. The components of the national plan include raising public awareness of acute cerebrovascular events, training specialized medical personnel, and establishing stroke units, as well as introducing quality measures for the treatment of acute cerebrovascular events in emergency departments and in hospitalizing wards [5]. In 2014, an Israeli National Stroke Registry (INSR) was established in the Israel Center for Disease Control (ICDC) [2]. The registry enables the identification of needs in the treatment and prevention of acute cerebrovascular events, the monitoring of changes in incidence rates and treatment quality, as well as the planning of interventions and the assessment of their efficacy [5].

During 2014–2016, the annual incidence rate of acute cerebrovascular events in the adult population (18+) in Israel was 3.3 cases per 1000 people or approximately 18,000 cases per year [6]. It is known that the incidence rate is higher among men, increases with age and, in Israel, higher among Arabs compared with Jews and others [6]. It is unknown whether the difference in the incidence rate across ethnicities emanates from a difference in the prevalence of modifiable risk factors (sedentary lifestyle, obesity, prevention and management of disorders and diseases like hypertension, dyslipidemia and diabetes), from a genetic predisposition, or from a combination of both.

There is some evidence indicating that the incidence rate of acute cerebrovascular events in developed countries had leveled off and has even been decreasing in the past decades. However, due to expected demographic changes in the size and composition of the population in these countries, and in particular the aging of the population, the number events and the associated economic burden is expected to increase in the following decades [4, 7–9].

In Israel, based on the observed trends of fertility and life expectancy in the past decades, it is projected that substantial demographic changes in the joint distribution of gender, age and ethnicity will take place. It is hypothesized that these changes will bring an increase in the incidence of acute cerebrovascular events. The objective of this study is to provide a forecast of the expected annual number of acute cerebrovascular events in Israel for the coming decades.

A forecast of the expected number of acute cerebrovascular events in Israel is a tool to help policymakers make judicious decisions about the volume of service provision, quantify the need for additional professionals and infrastructure, as well as to evaluate the future burden of care of acute cerebrovascular events and address the associated organizational and economic implications.

## Methods

The forecast of the annual number of acute cerebrovascular events in the adult population in Israel is based on data from the INSR and the long-term population projection of the Israeli CBS.

Beginning from January 2014, the INSR in the ICDC collects data from all the general hospitals in Israel about admitted adults (aged 18+) who had an acute cerebrovascular event [2]. The term ‘acute cerebrovascular event’ includes both ischemic and hemorrhagic strokes, as well as transient ischemic attack (case identification is the recorded diagnosis at discharge according to the International Classification of Disease, 9th edition), without distinction between a first or recurrent event. The registry includes both demographic and clinical data about the patient, the event and the treatment provided during hospitalization. For each event, the data we obtained from the INSR included date of birth, gender, ethnicity, date of admission and type of event. We obtained data about all acute stroke events reported from January 2014 until the end of the third quarter of 2017.

The Israeli CBS publishes annual updated population data [10]. These include data about the average size of various population subgroups defined by age, gender and ethnicity. In addition, periodically, the Israeli CBS publishes long-term population forecasts. Based on different assumptions about fertility, immigration and life expectancy, the CBS generates three alternative population forecasts (high, intermediate and low). We used the CBS population forecasts for 2015–2065 [11]. Although the CBS population forecast enables to generate a forecast until 2065, we decided to present a forecast until 2040, a time horizon that is more tangible for practical planning.

Whereas the INSR partitions the population to either ‘Jews’, ‘Arabs’ or ‘Others’, the CBS partitions the population to either ‘Jews and others’ or ‘Arabs’. Therefore, to generate a forecast of the number of acute cerebrovascular events, we used the finest possible joint partition, i.e., ‘Jews and others’ or ‘Arabs’.

We defined the age groups 18–39, 40–49, 50–59, 60–69, 70–79, 80–89, 90+. The partition to fine age categories was guided by two principal facts: first, the incidence of acute cerebrovascular events increases with age in an exponential pattern [4, 12]. Second, the life expectancy in Israel is high (80.2 years and 84.1 years for men and women, respectively, as of 2015) and the projected changes in the population age distribution emanate from substantial changes in the size of the older age groups.

For each year during 2014–2016, and for each subgroup, defined by gender, age group and ethnicity we calculated the subgroup-specific annual incidence rate of acute cerebrovascular event. We calculated the subgroup-specific mean annual incidence rate in these years. For each subgroup, the expected number of acute cerebrovascular events for each year was calculated as the product of the subgroup-specific mean annual incidence rate by the subgroup’s forecasted population size. The expected number of acute cerebrovascular events for each year was calculated as the sum of these products across all subgroups.

The CBS population forecast relies on specific assumptions about future mortality rates, projected from long-term historical data [11]. It is reasonable to hypothesize that the projected increase in life expectancy would be associated also with changes in the prevalence of risk factors for cardiovascular disease in general and for acute cerebrovascular events in particular. Hence, conforming with evidence in the literature about steady or even decreasing incidence rates in the past decades, a forecast, based on the assumption of constant annual incidence rates, should be considered as an upper bound for the future number of acute cerebrovascular events.

Information is scarce about projected changes in the incidence of acute cerebrovascular events and there is much uncertainty about the future prevalence of important risk factors for such events (e.g. the prevalence of diabetes, obesity, hypertension and unhealthy lifestyle) [13]. An important work from England (Oxford Vascular Study) studied the change in the annual incidence rate of acute cerebrovascular events [14]. First acute cerebrovascular events, which were diagnosed in hospital or in ambulatory care were included, without making distinction between different types of stroke. Similar registration and validation methods were employed in the same district, during two time periods 20 years apart. The authors reported a 27% decrease in the annual incidence rate of first acute cerebrovascular event between the early 1980’s and early 2000’s. These findings underlie the scenario assuming decreasing annual incidence rates at the rate of 7.25% every 5 years. The likelihood that the incidence rate of acute cerebrovascular events will continue to decrease at this rate in the future is low. Thus, a forecast under this assumption should be considered as a lower bound for the future number of acute cerebrovascular events.

A third, mid-way scenario, was generated assuming decreasing annual incidence rates at a rate of 2% every 5 years. The two latter assumptions about the expected trend of incidence rates of acute cerebrovascular events were also employed in a similar work from Sweden [8].

The forecasts were generated for each of the three alternative CBS population forecasts (low, intermediate and high) until 2040. The main text addresses the intermediate growth scenario, whereas forecasts for the low and high growth scenarios are included in Additional file 1 supplements C and D, respectively. Supplement E includes forecasts by event type (transient ischemic attack, ischemic stroke, hemorrhagic stroke), based on the intermediate population growth scenario.

Statistical analysis and tests were conducted using SPSS. The forecasts and the figures were generated using EXCEL.

The study was a joint project of the Medical Directorate and the ICDC and it was conducted under the auspice of the National Plan for the Treatment and Prevention of Stroke Damage. It was therefore exempted from approval of the MOH ethics committee.

## Results

### Subgroup-specific mean annual incidence rates of acute cerebrovascular events during 2014–2016

From January 2014 until the end of the third quarter of 2017, 68,126 cases of acute cerebrovascular events in adults were admitted to hospitals and were reported to the INSR. Some patients had more than one event during this time period: 53,246 patients (89% of all patients) had a single event, 5553 (9%) had two events and 1144 (2%) had more than two events. The annual number of acute cerebrovascular events was similar during 2014–2017 (Table 1).

**Table 1:** Acute cerebrovascular events in Israel, adult population (18+), from 2014 to the third quarter of 2017

Total Number of events (*N*)	68,126
By year (*N*)	
2014	17,504
2015	18,494
2016	18,517
2017 (Q1-Q3)	13,611
By type (%)	
Hemorrhagic stroke	7.2
Ischemic stroke	64.7
Transient ischemic attack	28.1
By gender (%)	
Women	46.1
Men	53.9
By population group (%)	
Jews	81.2
Others	3.8
Arabs	15

INSR data, Q = quarter

The annual incidence rate was higher as the population was older, it was higher among men than among women, and higher among Arabs, for both genders and across all age groups but Arabs over 80 years old, among whom, women had a higher incidence rate than men and, at 90+, both genders had lower incidence rates compared to Jews and Others (Table 2, Additional file 1: Table S1).

**Table 2:** Subgroup-specific mean annual incidence rate of acute cerebrovascular events in Israel during 2014–2016, adult population (18+) (number of cases per 1000)

Age group	Jews and Others		Arabs	
	Men	Women	Men	Women
18–39	0.10	0.12	0.17	0.12
40–49	0.87	0.52	1.65	0.85
50–59	3.38	1.67	5.96	3.30
60–69	7.74	3.85	13.49	7.54
70–79	14.74	9.90	21.80	17.54
80–89	25.06	22.30	27.90	30.37
90+	33.32	31.87	24.58	25.00

INSR data

### Israeli CBS population forecast for the intermediate population growth scenario

**Fig. 1:**
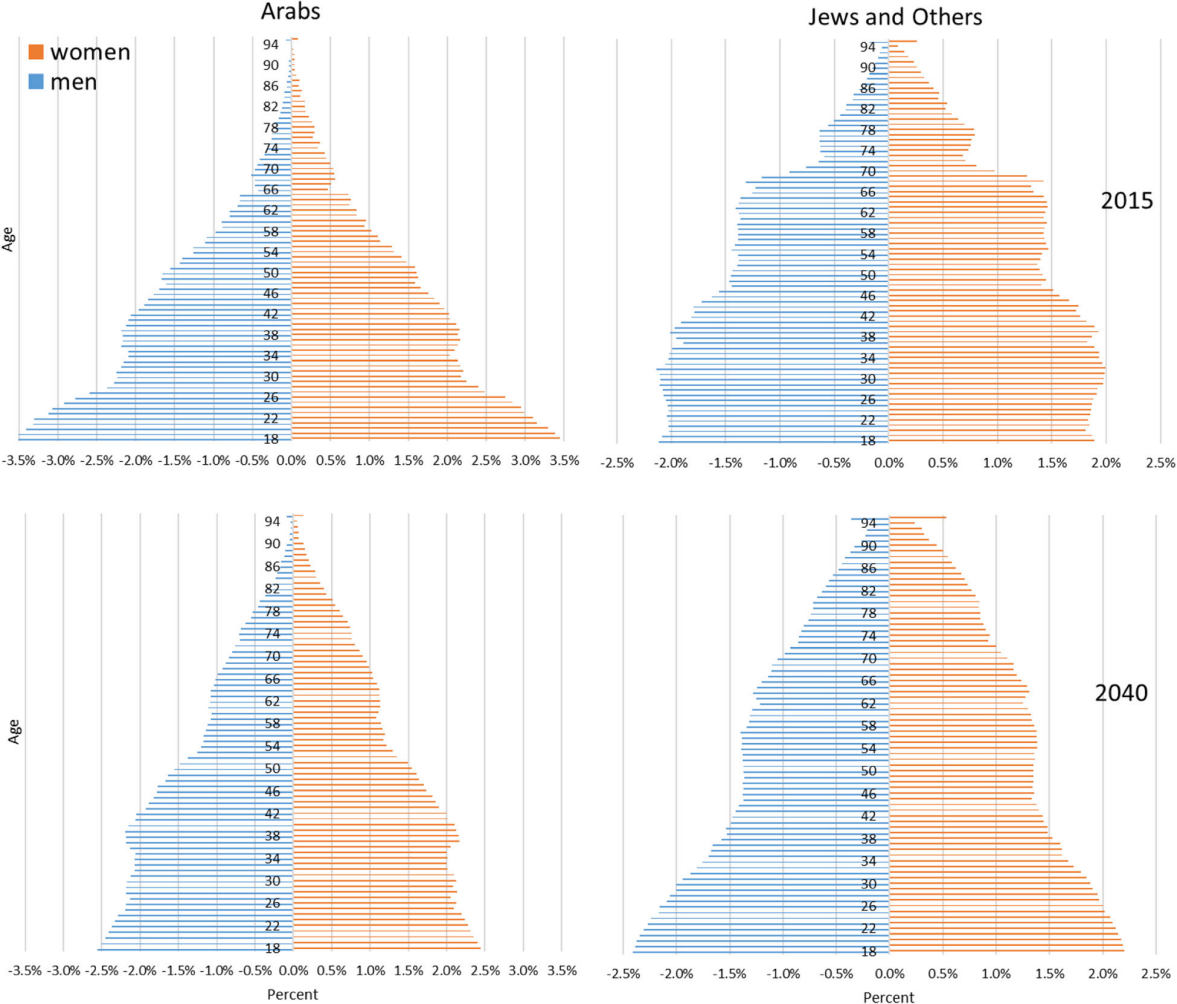
Israel’s adult (18+) population distribution by age and gender, intermediate growth scenario 2015 and 2040, by ethnicity

By 2040 the adult population (18+) in Israel is expected to increase 1.6-fold. The proportion of the Arab adult population is expected to increase from 18 to 21%. The proportion of men 70+ is expected to increase from 9 to 14%, and the proportion of women 70+ is expected to increase from 12 to 17%. The proportion of men 80+ is expected to increase from 3 to 6% and that of women 80+ from 5 to 8% (Fig. 1, Additional file 1: Table S2).

### A forecast of the annual number of acute cerebrovascular events 2015–2040 for the intermediate population growth scenario

**Fig. 2:**
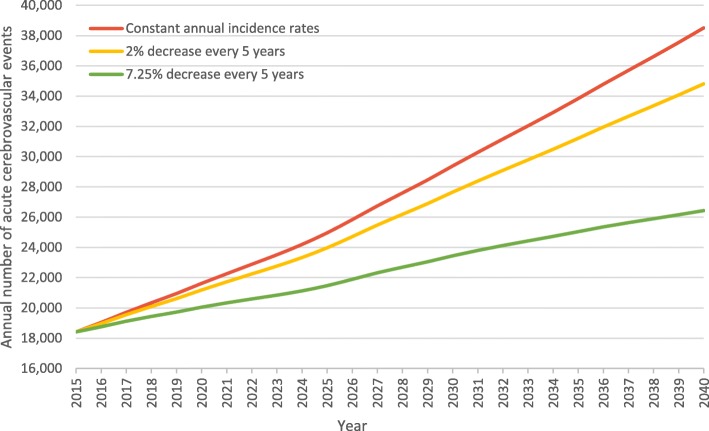
Forecast of the annual number of acute cerebrovascular events within the adult population (18+) in Israel, intermediate growth scenario 2015–2040, under various assumptions about expected change in incidence rate

Based on the CBS intermediate population growth scenario, assuming constant incidence rates across all population subgroups, by 2040 the annual number of acute cerebrovascular events within the adult population is expected to increase from 18,400 to 38,500 events (annual incidence rate: 4.3 per 1000). Under decreasing incidence rates, assuming a 2% decrease every 5 years, by 2040 the annual number of acute cerebrovascular events is expected to increase to 34,800 (annual incidence rate: 3.9 per 1000), and to 26,400 assuming a 7.25% decrease every 5 years (annual incidence rate: 3.0 per 1000) (Fig. 2, Additional file 1: Tables S2-S5).

By 2040, the population of Jews and others is expected to increase 1.5-fold and the annual number of acute cerebrovascular events in this population is expected to increase 1.9-fold, assuming constant incidence rates, or 1.8-fold and 1.3-fold, assuming decreasing incidence rates at a rate of 2% every 5 years and 7.25% every 5 years, respectively (Additional file 1: Tables S2-S5).

The Arab population is expected to increase 1.8-fold and the annual number of acute cerebrovascular events is expected to increase 2.9-fold in this ethnic group, assuming constant incidence rates, or 2.7-fold and 2.0-fold, assuming decreasing incidence rates at a rate of 2% every 5 years and 7.25% every 5 years, respectively (Additional file 1: Tables S2-S5).

Accordingly, whereas presently, events in Arab patients account for 15% of acute cerebrovascular events admitted to hospitals, by 2040 Arab patients will account for more than 21% of the events (irrespective of the underlying assumption on annual incidence rate).

The greatest proportional increase in the annual number of acute cerebrovascular events is expected within the older age groups: the size of the population age groups 70–79, 80–89 and 90+ is expected to increase 2.1, 2.5 and 3.1-fold, respectively. The annual number of acute cerebrovascular events within these subgroups is expected to increase 2.2, 2.5 and 3.1-fold, respectively, assuming constant incidence rates; 2.0, 2.3 and 2.8-fold, respectively, assuming decreasing incidence rates at a constant rate of 2% every 5 years; and, 1.5, 1.7 and 2.1-fold, respectively, assuming decreasing incidence rates at a constant rate of 7.25% every 5 years (Additional file 1: Tables S2-S5).

Accordingly, whereas presently, events in patients over 70 account for 59% of acute cerebrovascular events admitted to hospitals, and patients over 80 account for 33%, by 2040 patients over 70 will account for 68% of the events, and patients over 80 will account for 42% of the events (irrespective of the underlying assumption on the annual incidence rate).

## Discussion

The number of acute cerebrovascular events in Israel is expected to increase substantially in the following decades. The expected increase is attributed to demographic changes in the size and composition of the adult population and, in particular, to a significant rightward shift in the adult population age distribution and to an absolute growth of the Arab population in Israel.

The expected increase in the number of events and the changes in the age distribution of adults affected by acute cerebrovascular events have implications on provision of both acute and long-term care: the prevalence of co-morbidities is higher among older adults, and acute administration of reperfusion treatments for ischemic stroke in these patients, pharmacological or endovascular, is associated with a higher risk of complications. The prognosis of older adults after an acute cerebrovascular event is worse, requiring complex and prolonged rehabilitation and long-term care. A greater proportion of patients will require long-term care services. It is thus important to assess the availability and quality of long-term care services for acute cerebrovascular event patients, including nursing home care and rehabilitation services. Because of the high incidence rate within the Arab population, compared to the Jewish population, the expected absolute growth of this population significantly contributes to the expected increase in the number of acute cerebrovascular events. Regardless of whether the higher incidence rate among Arabs emanates from a higher prevalence of risk factors, from a genetic predisposition, or from a combination of both, it is important to ensure that the Arab population has access to high quality care services, to increase its awareness and knowledge about acute cerebrovascular event, and to employ appropriate prevention strategies.

Alongside these changes, the economic burden associated with acute cerebrovascular events is expected to increase due to increases in the volumes of both acute hospital care and community-based intermediate and long-term care. Thus, it is of great importance to plan and implement effective programs for the prevention of acute cerebrovascular events. The literature shows that much of the burden associated with cerebrovascular events can be attributed to modifiable atherosclerotic risk factors [4]. A recently published work delineates an action plan for stroke in Europe, emphasizing primary and secondary prevention, among which are policies that target risk factors modification (metabolic, behavioral and environmental) by means of pharmacological and non-pharmacological interventions, at the primary care level, as well as at the population level, through legislative changes, media campaigns and educational and preventive measures in schools, workplace and the community [3].

The types of acute cerebrovascular events differ in terms of risk factors, severity, prognosis and treatment. The timeline and the initial management of the different event types, from symptom occurrence until after brain imaging in the hospital, is very similar; therefore the infrastructure of the emergency services that is required to manage an acute cerebrovascular event is similar for the different event types (transient ischemic attacks – diagnosed prior to or upon admission, based on the timing of appearance and resolution of symptoms, for which the work-up may be somewhat different, both in terms of urgency and the type of evaluation – may be an exception). Thus, a forecast of the number of acute cerebrovascular events of any type is an appropriate tool for the purpose of planning and improving acute care services. However, the requirements for the intermediate inpatient care and long term ambulatory care services following different event types (transient ischemic attack, ischemic stroke or hemorrhagic stroke) may be quite different in terms of intensity and duration. The mean length of stay of inpatients diagnosed with transient ischemic attack, ischemic stroke or hemorrhagic stroke was 3.8, 8.4 and 13.4 days, respectively. Further, as mentioned above, the prognosis for the different event types varies considerably: the 30-day mortality rate was < 1% for transient ischemic attack, 10% following ischemic stroke and 30% following hemorrhagic stroke and 1-year mortality rates were 7, 23 and 43% respectively [6]. Forecasts of the number of events for each of the event types based on the intermediate population growth scenario are included in Additional file 1 supplement D. These forecasts may aid the planning and organization of intermediate and long-term services for post-event patients. The aforementioned action plan for stroke provides guidelines about secondary therapy, rehabilitation and life after stroke [3].

### Study limitations

The INSR collects data about acute cerebrovascular events in adults admitted to general hospitals in Israel, without distinguishing between first or recurrent events. Adults who experienced an event and did not seek treatment in hospital were not included in the INSR data. Such events may include mild, transient ischemic attacks, but also severe events which were not referred to hospital treatment (e.g. in care facilities or hospices) or that were associated with death prior to any medical treatment. The MOH estimate is that, presently, additional 2000 acute cerebrovascular events not admitted to hospital occur annually [5]. Thus, the annual incidence rates upon which the forecasts are generated may be an underestimate, from an epidemiological perspective.

In this work we generated forecasts until 2040. The chance that these long-term forecast will turn to be accurate in the future is low. It should be emphasized, as the authors of the CBS population forecast do, that there are interrelations between the population forecast and the planning agencies, and this remark is also applicable to the forecast provided in this work [11]. Nevertheless, the different scenarios of the CBS forecast provide a reliable range for the future population size and composition in the long term, which is essential and sufficient for planning purposes. The forecasts in this work thus provide a reliable estimated range of the future number of acute cerebrovascular events in Israel, cardinally determined by the size and the composition of the population, an important input for planning health policies and services that aim to improve acute cerebrovascular event care. Similar works in the literature provide long term forecasts for similar purposes [8, 12, 13, 15]. These forecasts (including ours) were generated under certain assumptions about the trend of the annual incidence rate of acute cerebrovascular events, which were applied uniformly across the different population subgroups. These assumptions are rather simplistic whereas more sophisticated scenarios, assuming variable changes in the prevalence of risk factors for acute cerebrovascular events across subpopulations, are possible.

## Conclusions

The number of acute cerebrovascular events in Israel is expected to increase substantially in the following decades. In order to ensure both high-quality care and equity, it is imperative to evaluate the preparedness of short- and long-term services for acute cerebrovascular event care and their geographical deployment thereof. It is also of great importance to plan and implement effective programs for the prevention of acute cerebrovascular events.

## Additional file

Additional file 1: **Table S1.** Subgroup-specific annual incidence rate of acute cerebrovascular events in Israel, 2014–2016, by gender, ethnicity and age group (number of cases per 1000). **Table S2.** Population forecast, intermediate population growth scenario 2015–2040. **Table S3.** Forecast of the annual number of acute cerebrovascular events in the adult population (18+) in Israel, intermediate population growth scenario 2015–2040, assuming constant annual incidence rates. **Table S4.** forecast of the annual number of acute cerebrovascular events in the adult population (18+) in Israel, intermediate population growth scenario 2015–2040, assuming decreasing annual incidence rates at a rate of 2% every 5 years. **Table S5.** forecast of the annual number of acute cerebrovascular events in the adult population (18+) in Israel, intermediate population growth scenario 2015–2040, assuming decreasing annual incidence rates at a rate of 7.25% every 5 years. **Table S6.** Population forecast, low population growth scenario 2015–2040. **Table S7.** forecast of the annual number of acute cerebrovascular events in the adult population (18+) in Israel, low population growth scenario 2015–2040, assuming constant annual incidence rates. **Table S8.** forecast of the annual number of acute cerebrovascular events in the adult population (18+) in Israel, low population growth scenario 2015–2040, assuming decreasing annual incidence rates at a rate of 2% every 5 years. **Table S9.** forecast of the annual number of acute cerebrovascular events in the adult population (18+) in Israel, low population growth scenario 2015–2040, assuming decreasing annual incidence rates at a rate of 7.25% every 5 years. **Table S10.** Population forecast, high population growth scenario 2015–2040. **Table S11.** forecast of the annual number of acute cerebrovascular events in the adult population (18+) in Israel, high population growth scenario 2015–2040, assuming constant annual incidence rates. **Table S12.** forecast of the annual number of acute cerebrovascular events in the adult population (18+) in Israel, high population growth scenario 2015–2040, assuming decreasing annual incidence rates at a rate of 2% every 5 years. **Table S13.** forecast of the annual number of acute cerebrovascular events in the adult population (18+) in Israel, high population growth scenario 2015–2040, assuming decreasing annual incidence rates at a rate of 7.25% every 5 years. **Figure S1.** Forecast of the annual number of acute cerebrovascular events within the adult population (18+), for alternative population scenarios 2015–2040, under various assumption about stroke incidence rate. **Table S14.** Subgroup-specific mean annual incidence rate of acute cerebrovascular events in Israel during 2014–2016, by gender, ethnicity, age group and type of event (number of cases per 1000). **Table S15.** forecast of the annual number of acute cerebrovascular events in the adult population (18+) in Israel, intermediate population growth scenario 2015–2040, assuming constant annual incidence rates, by event type, gender and ethnicity. **Table S16.** forecast of the annual number of acute cerebrovascular events in the adult population (18+) in Israel, intermediate population growth scenario 2015–2040, assuming constant annual incidence rates, by event type and age group. **Table S17.** forecast of the annual number of acute cerebrovascular events in the adult population (18+) in Israel, intermediate population growth scenario 2015–2040, assuming decreasing annual incidence rates at a rate of 2% every 5 years, by event type, gender and ethnicity. **Table S18.** forecast of the annual number of acute cerebrovascular events in the adult population (18+) in Israel, intermediate population growth scenario 2015–2040, assuming decreasing annual incidence rates at a rate of 2% every 5 years, by event type and age group. **Table S19.** forecast of the annual number of acute cerebrovascular events in the adult population (18+) in Israel, intermediate population growth scenario 2015–2040, assuming decreasing annual incidence rates at a rate of 7.25% every 5 years, by event type, gender and ethnicity. **Table S20.** forecast of the annual number of acute cerebrovascular events in the adult population (18+) in Israel, intermediate population growth scenario 2015–2040, assuming decreasing annual incidence rates at a rate of 7.25% every 5 years, by event type and age group. **Figure S2.** Forecast of the annual number of acute cerebrovascular events within the adult population, 2015–2040, intermediate population growth scenario, under various assumption about stroke incidence rate, by type of event.

## Data Availability

Israel CBS population forecast for 2015–2065 is publicly available online (Accessed May 6, 2019) https://www.cbs.gov.il; INSR data is available from the ICDC in Israel MOH.

## Abbreviations

CBS: Central bureau of statistics
ICDC: Israeli center for disease control
INSR: National stroke registry
MOH: Ministry of health
