# Supplementary material for: The number of acute cerebrovascular events in Israel: a forecast until 2040
Source: Isr J Health Policy Res. 2019 Oct 1;8:67. doi: 10.1186/s13584-019-0337-1 (PMC6771084; doi:10.1186/s13584-019-0337-1)
Supplement: Supplementary file 1 — Table S1. Subgroup-specific annual incidence rate of acute cerebrovascular events in Israel, 2014–2016, by gender, ethnicity and age group (number of cases per 1000). Table S2. Population forecast, intermediate population growth scenario 2015–2040. Table S3. Forecast of the annual number of acute cerebrovascular events in the adult population (18+) in Israel, intermediate population growth scenario 2015–2040, assuming constant annual incidence rates. Table S4. forecast of the annual number of acute cerebrovascular events in the adult population (18+) in Israel, intermediate population growth scenario 2015–2040, assuming decreasing annual incidence rates at a rate of 2% every 5 years. Table S5. forecast of the annual number of acute cerebrovascular events in the adult population (18+) in Israel, intermediate population growth scenario 2015–2040, assuming decreasing annual incidence rates at a rate of 7.25% every 5 years. Table S6. Population forecast, low population growth scenario 2015–2040. Table S7. forecast of the annual number of acute cerebrovascular events in the adult population (18+) in Israel, low population growth scenario 2015–2040, assuming constant annual incidence rates. Table S8. forecast of the annual number of acute cerebrovascular events in the adult population (18+) in Israel, low population growth scenario 2015–2040, assuming decreasing annual incidence rates at a rate of 2% every 5 years. Table S9. forecast of the annual number of acute cerebrovascular events in the adult population (18+) in Israel, low population growth scenario 2015–2040, assuming decreasing annual incidence rates at a rate of 7.25% every 5 years. Table S10. Population forecast, high population growth scenario 2015–2040. Table S11. forecast of the annual number of acute cerebrovascular events in the adult population (18+) in Israel, high population growth scenario 2015–2040, assuming constant annual incidence rates. Table S12. forecast of the annual number of acute cerebrovasc [file 13584_2019_337_MOESM1_ESM.docx]

**Supplements**

1. Subgroup-specific annual incidence rate of acute cerebrovascular events, 2014-2016

Table S1: Subgroup-specific annual incidence rate of acute cerebrovascular events in Israel, 2014-2016, by gender, ethnicity and age group (number of cases per 1,000)

| 2014 | | | | | |
| --- | --- | --- | --- | --- | --- |
|  | Jews and Others | | Arabs | | Total |
| Age group | Men | Women | Men | Women |  |
| 18-39 | 0.10 | 0.12 | 0.19 | 0.14 | 0.12 |
| 40-49 | 0.93 | 0.49 | 1.55 | 0.74 | 0.79 |
| 50-59 | 3.55 | 1.60 | 5.84 | 3.18 | 2.85 |
| 60-69 | 7.48 | 3.72 | 13.77 | 6.90 | 5.95 |
| 70-79 | 14.89 | 9.78 | 21.69 | 16.70 | 12.71 |
| 80-89 | 24.46 | 22.43 | 28.18 | 29.19 | 23.55 |
| 90+ | 33.67 | 32.80 | 32.50 | 22.50 | 32.81 |
| Total | 3.66 | 3.01 | 2.94 | 2.10 | 3.18 |
| 2015 | | | | | |
|  | Jews and Others | | Arabs | | Total |
| Age group | Men | Women | Men | Women |  |
| 18-39 | 0.11 | 0.12 | 0.19 | 0.13 | 0.12 |
| 40-49 | 0.83 | 0.55 | 1.62 | 0.80 | 0.79 |
| 50-59 | 3.35 | 1.82 | 6.06 | 3.39 | 2.91 |
| 60-69 | 8.02 | 4.09 | 12.54 | 7.72 | 6.34 |
| 70-79 | 14.82 | 10.46 | 21.77 | 16.51 | 13.04 |
| 80-89 | 26.17 | 22.56 | 26.30 | 28.31 | 24.20 |
| 90+ | 32.73 | 31.23 | 22.50 | 23.33 | 31.38 |
| Total | 3.78 | 3.16 | 2.93 | 2.21 | 3.30 |
| 2016 | | | | | |
|  | Jews and Others | | Arabs | | Total |
| Age group | Men | Women | Men | Women |  |
| 18-39 | 0.09 | 0.12 | 0.14 | 0.11 | 0.11 |
| 40-49 | 0.84 | 0.54 | 1.78 | 1.02 | 0.83 |
| 50-59 | 3.23 | 1.60 | 5.98 | 3.33 | 2.78 |
| 60-69 | 7.71 | 3.74 | 14.16 | 7.99 | 6.14 |
| 70-79 | 14.52 | 9.46 | 21.93 | 19.40 | 12.59 |
| 80-89 | 24.54 | 21.92 | 29.20 | 33.62 | 23.51 |
| 90+ | 33.54 | 31.57 | 18.75 | 29.17 | 31.96 |
| Total | 3.69 | 3.01 | 3.10 | 2.48 | 3.24 |

INSR data and CBS annual statistical reports

1. Forecast based on CBS intermediate population growth scenario

Table S2: Population forecast, intermediate population growth scenario 2015-2040

|  | 2015 | 2020 | 2025 | 2030 | 2035 | 2040 |
| --- | --- | --- | --- | --- | --- | --- |
| Total | 5,665 | 6,203 | 6,790 | 7,448 | 8,156 | 8,917 |
| by gender and ethnicity |  |  |  |  |  |  |
| Arab men | 521 | 611 | 697 | 782 | 868 | 960 |
| Arab women | 519 | 606 | 689 | 772 | 855 | 943 |
| Jewish and other men | 2,240 | 2,423 | 2,633 | 2,879 | 3,152 | 3,446 |
| Jewish and other women | 2,385 | 2,564 | 2,771 | 3,015 | 3,282 | 3,567 |
| by age group |  |  |  |  |  |  |
| 18-39 | 2,600 | 2,765 | 2,969 | 3,247 | 3,576 | 3,932 |
| 40-49 | 971 | 1,089 | 1,152 | 1,200 | 1,250 | 1,339 |
| 50-59 | 786 | 843 | 969 | 1,086 | 1,150 | 1,200 |
| 60-69 | 700 | 740 | 766 | 824 | 949 | 1,065 |
| 70-79 | 368 | 488 | 627 | 669 | 700 | 760 |
| 80-89 | 195 | 223 | 244 | 343 | 442 | 482 |
| 90+ | 45 | 54 | 63 | 78 | 90 | 140 |

Faran and Klinger 2018

Table S3: Forecast of the annual number of acute cerebrovascular events in the adult population (18+) in Israel, intermediate population growth scenario 2015-2040, assuming constant annual incidence rates

|  | 2015 | 2020 | 2025 | 2030 | 2035 | 2040 |
| --- | --- | --- | --- | --- | --- | --- |
| Total | 18,415 | 21,616 | 24,958 | 29,380 | 33,835 | 38,509 |
| by gender and ethnicity |  |  |  |  |  |  |
| Arab men | 1,571 | 1,986 | 2,510 | 3,103 | 3,793 | 4,545 |
| Arab women | 1,184 | 1,479 | 1,878 | 2,337 | 2,913 | 3,554 |
| Jewish and other men | 8,341 | 9,735 | 11,125 | 12,864 | 14,537 | 16,235 |
| Jewish and other women | 7,318 | 8,417 | 9,444 | 11,076 | 12,591 | 14,175 |
| by age group |  |  |  |  |  |  |
| 18-39 | 307 | 329 | 354 | 387 | 425 | 465 |
| 40-49 | 783 | 877 | 925 | 972 | 1,036 | 1,131 |
| 50-59 | 2,243 | 2,455 | 2,831 | 3,171 | 3,349 | 3,523 |
| 60-69 | 4,301 | 4,649 | 4,956 | 5,440 | 6,283 | 7,051 |
| 70-79 | 4,711 | 6,239 | 8,017 | 8,704 | 9,296 | 10,235 |
| 80-89 | 4,624 | 5,320 | 5,852 | 8,214 | 10,587 | 11,623 |
| 90+ | 1,445 | 1,747 | 2,023 | 2,493 | 2,859 | 4,480 |

## Calculation based on INSR data and Faran and Klinger 2018

Table S4: forecast of the annual number of acute cerebrovascular events in the adult population (18+) in Israel, intermediate population growth scenario 2015-2040, assuming decreasing annual incidence rates at a rate of 2% every 5 years

|  | 2015 | 2020 | 2025 | 2030 | 2035 | 2040 |
| --- | --- | --- | --- | --- | --- | --- |
| Total | 18,415 | 21,184 | 23,970 | 27,652 | 31,208 | 34,809 |
| by gender and ethnicity |  |  |  |  |  |  |
| Arab men | 1,571 | 1,946 | 2,411 | 2,921 | 3,498 | 4,108 |
| Arab women | 1,184 | 1,449 | 1,804 | 2,199 | 2,687 | 3,212 |
| Jewish and other men | 8,341 | 9,540 | 10,685 | 12,107 | 13,409 | 14,675 |
| Jewish and other women | 7,318 | 8,248 | 9,070 | 10,425 | 11,614 | 12,813 |
| by age group |  |  |  |  |  |  |
| 18-39 | 307 | 322 | 340 | 364 | 392 | 420 |
| 40-49 | 783 | 860 | 889 | 915 | 956 | 1,023 |
| 50-59 | 2,243 | 2,406 | 2,718 | 2,984 | 3,089 | 3,184 |
| 60-69 | 4,301 | 4,556 | 4,759 | 5,120 | 5,795 | 6,374 |
| 70-79 | 4,711 | 6,115 | 7,700 | 8,192 | 8,574 | 9,252 |
| 80-89 | 4,624 | 5,214 | 5,620 | 7,731 | 9,765 | 10,507 |
| 90+ | 1,445 | 1,712 | 1,943 | 2,346 | 2,637 | 4,049 |

## Calculation based on INSR data and Faran and Klinger 2018

Table S5: forecast of the annual number of acute cerebrovascular events in the adult population (18+) in Israel, intermediate population growth scenario 2015-2040, assuming decreasing annual incidence rates at a rate of 7.25% every 5 years

|  | 2015 | 2020 | 2025 | 2030 | 2035 | 2040 |
| --- | --- | --- | --- | --- | --- | --- |
| Total | 18,415 | 20,049 | 21,470 | 23,442 | 25,039 | 26,432 |
| by gender and ethnicity |  |  |  |  |  |  |
| Arab men | 1,571 | 1,842 | 2,160 | 2,476 | 2,807 | 3,120 |
| Arab women | 1,184 | 1,371 | 1,616 | 1,865 | 2,156 | 2,439 |
| Jewish and other men | 8,341 | 9,029 | 9,571 | 10,264 | 10,758 | 11,144 |
| Jewish and other women | 7,318 | 7,806 | 8,124 | 8,837 | 9,318 | 9,729 |
| by age group |  |  |  |  |  |  |
| 18-39 | 307 | 305 | 305 | 309 | 314 | 319 |
| 40-49 | 783 | 813 | 796 | 775 | 767 | 776 |
| 50-59 | 2,243 | 2,277 | 2,435 | 2,530 | 2,478 | 2,418 |
| 60-69 | 4,301 | 4,311 | 4,263 | 4,341 | 4,650 | 4,840 |
| 70-79 | 4,711 | 5,787 | 6,897 | 6,944 | 6,879 | 7,025 |
| 80-89 | 4,624 | 4,934 | 5,034 | 6,554 | 7,835 | 7,978 |
| 90+ | 1,445 | 1,621 | 1,740 | 1,989 | 2,116 | 3,075 |

## Calculation based on INSR data and Faran and Klinger 2018

1. Forecast based on CBS low population growth scenario

Table S6: Population forecast, low population growth scenario 2015-2040

|  | 2015 | 2020 | 2025 | 2030 | 2035 | 2040 |
| --- | --- | --- | --- | --- | --- | --- |
| Total | 5,665 | 6,187 | 6,747 | 7,373 | 8,026 | 8,663 |
| by gender and ethnicity |  |  |  |  |  |  |
| Arab men | 521 | 610 | 694 | 777 | 857 | 936 |
| Arab women | 519 | 605 | 687 | 767 | 845 | 922 |
| Jewish and other men | 2,240 | 2,416 | 2,615 | 2,847 | 3,097 | 3,342 |
| Jewish and other women | 2,385 | 2,556 | 2,751 | 2,981 | 3,226 | 3,463 |
| by age group |  |  |  |  |  |  |
| 18-39 | 2,600 | 2,765 | 2,967 | 3,244 | 3,556 | 3,829 |
| 40-49 | 971 | 1,088 | 1,151 | 1,198 | 1,247 | 1,335 |
| 50-59 | 786 | 842 | 967 | 1,082 | 1,144 | 1,192 |
| 60-69 | 700 | 738 | 760 | 815 | 936 | 1,048 |
| 70-79 | 368 | 484 | 613 | 648 | 672 | 725 |
| 80-89 | 195 | 218 | 232 | 318 | 399 | 427 |
| 90+ | 45 | 52 | 57 | 66 | 72 | 108 |

Faran and Klinger 2018

Table S7: forecast of the annual number of acute cerebrovascular events in the adult population (18+) in Israel, low population growth scenario 2015-2040, assuming constant annual incidence rates

|  | 2015 | 2020 | 2025 | 2030 | 2035 | 2040 |
| --- | --- | --- | --- | --- | --- | --- |
| Total | 18,415 | 21,355 | 24,253 | 28,050 | 31,747 | 35,498 |
| by gender and ethnicity |  |  |  |  |  |  |
| Arab men | 1,571 | 1,975 | 2,473 | 3,021 | 3,651 | 4,322 |
| Arab women | 1,184 | 1,466 | 1,833 | 2,248 | 2,768 | 3,341 |
| Jewish and other men | 8,341 | 9,624 | 10,825 | 12,296 | 13,649 | 14,970 |
| Jewish and other women | 7,318 | 8,290 | 9,122 | 10,485 | 11,680 | 12,865 |
| by age group |  |  |  |  |  |  |
| 18-39 | 307 | 328 | 354 | 387 | 422 | 453 |
| 40-49 | 783 | 877 | 924 | 970 | 1,033 | 1,127 |
| 50-59 | 2,243 | 2,453 | 2,824 | 3,157 | 3,330 | 3,497 |
| 60-69 | 4,301 | 4,630 | 4,916 | 5,377 | 6,194 | 6,930 |
| 70-79 | 4,711 | 6,181 | 7,840 | 8,414 | 8,908 | 9,748 |
| 80-89 | 4,624 | 5,208 | 5,563 | 7,621 | 9,561 | 10,289 |
| 90+ | 1,445 | 1,678 | 1,832 | 2,123 | 2,299 | 3,453 |

## Calculation based on INSR data and Faran and Klinger 2018

Table S8: forecast of the annual number of acute cerebrovascular events in the adult population (18+) in Israel, low population growth scenario 2015-2040, assuming decreasing annual incidence rates at a rate of 2% every 5 years

|  | 2015 | 2020 | 2025 | 2030 | 2035 | 2040 |
| --- | --- | --- | --- | --- | --- | --- |
| Total | 18,415 | 20,928 | 23,292 | 26,401 | 29,282 | 32,087 |
| by gender and ethnicity |  |  |  |  |  |  |
| Arab men | 1,571 | 1,935 | 2,375 | 2,844 | 3,367 | 3,907 |
| Arab women | 1,184 | 1,437 | 1,760 | 2,116 | 2,553 | 3,020 |
| Jewish and other men | 8,341 | 9,431 | 10,396 | 11,573 | 12,589 | 13,532 |
| Jewish and other women | 7,318 | 8,124 | 8,761 | 9,868 | 10,773 | 11,629 |
| by age group |  |  |  |  |  |  |
| 18-39 | 307 | 322 | 340 | 364 | 389 | 409 |
| 40-49 | 783 | 859 | 888 | 913 | 953 | 1,019 |
| 50-59 | 2,243 | 2,404 | 2,712 | 2,972 | 3,071 | 3,161 |
| 60-69 | 4,301 | 4,538 | 4,722 | 5,061 | 5,713 | 6,264 |
| 70-79 | 4,711 | 6,057 | 7,530 | 7,919 | 8,217 | 8,811 |
| 80-89 | 4,624 | 5,104 | 5,343 | 7,173 | 8,819 | 9,301 |
| 90+ | 1,445 | 1,644 | 1,759 | 1,999 | 2,120 | 3,122 |

Calculation based on INSR data and Faran and Klinger 2018

Table S9: forecast of the annual number of acute cerebrovascular events in the adult population (18+) in Israel, low population growth scenario 2015-2040, assuming decreasing annual incidence rates at a rate of 7.25% every 5 years

|  | 2015 | 2020 | 2025 | 2030 | 2035 | 2040 |
| --- | --- | --- | --- | --- | --- | --- |
| Total | 18,415 | 19,806 | 20,864 | 22,381 | 23,494 | 24,365 |
| by gender and ethnicity |  |  |  |  |  |  |
| Arab men | 1,571 | 1,831 | 2,127 | 2,411 | 2,702 | 2,967 |
| Arab women | 1,184 | 1,360 | 1,576 | 1,794 | 2,048 | 2,293 |
| Jewish and other men | 8,341 | 8,926 | 9,312 | 9,811 | 10,101 | 10,275 |
| Jewish and other women | 7,318 | 7,689 | 7,848 | 8,366 | 8,643 | 8,830 |
| by age group |  |  |  |  |  |  |
| 18-39 | 307 | 305 | 304 | 309 | 312 | 311 |
| 40-49 | 783 | 813 | 795 | 774 | 765 | 774 |
| 50-59 | 2,243 | 2,275 | 2,429 | 2,519 | 2,464 | 2,400 |
| 60-69 | 4,301 | 4,295 | 4,229 | 4,290 | 4,584 | 4,756 |
| 70-79 | 4,711 | 5,733 | 6,745 | 6,714 | 6,592 | 6,691 |
| 80-89 | 4,624 | 4,830 | 4,786 | 6,081 | 7,075 | 7,063 |
| 90+ | 1,445 | 1,556 | 1,576 | 1,694 | 1,701 | 2,370 |

## Calculation based on INSR data and Faran and Klinger 2018

1. Forecast based on CBS high population growth scenario

Table S10: Population forecast, high population growth scenario 2015-2040

|  | 2015 | 2020 | 2025 | 2030 | 2035 | 2040 |
| --- | --- | --- | --- | --- | --- | --- |
| Total | 5,665 | 6,218 | 6,828 | 7,515 | 8,272 | 9,150 |
| by gender and ethnicity |  |  |  |  |  |  |
| Arab men | 521 | 612 | 699 | 787 | 877 | 981 |
| Arab women | 519 | 607 | 692 | 776 | 863 | 962 |
| Jewish and other men | 2,240 | 2,429 | 2,649 | 2,907 | 3,201 | 3,543 |
| Jewish and other women | 2,385 | 2,570 | 2,788 | 3,045 | 3,332 | 3,664 |
| by age group |  |  |  |  |  |  |
| 18-39 | 2,600 | 2,766 | 2,970 | 3,249 | 3,595 | 4,032 |
| 40-49 | 971 | 1,089 | 1,153 | 1,202 | 1,252 | 1,342 |
| 50-59 | 786 | 844 | 971 | 1,090 | 1,155 | 1,205 |
| 60-69 | 700 | 743 | 771 | 832 | 960 | 1,079 |
| 70-79 | 368 | 492 | 638 | 687 | 722 | 787 |
| 80-89 | 195 | 228 | 255 | 365 | 480 | 530 |
| 90+ | 45 | 57 | 69 | 90 | 108 | 174 |

## Faran and Klinger 2018

Table S11: forecast of the annual number of acute cerebrovascular events in the adult population (18+) in Israel, high population growth scenario 2015-2040, assuming constant annual incidence rates

|  | 2015 | 2020 | 2025 | 2030 | 2035 | 2040 |
| --- | --- | --- | --- | --- | --- | --- |
| Total | 18,415 | 21,868 | 25,617 | 30,606 | 35,754 | 41,305 |
| by gender and ethnicity |  |  |  |  |  |  |
| Arab men | 1,571 | 1,999 | 2,549 | 3,177 | 3,920 | 4,746 |
| Arab women | 1,184 | 1,493 | 1,917 | 2,409 | 3,030 | 3,738 |
| Jewish and other men | 8,341 | 9,839 | 11,406 | 13,392 | 15,364 | 17,421 |
| Jewish and other women | 7,318 | 8,536 | 9,746 | 11,627 | 13,439 | 15,400 |
| by age group |  |  |  |  |  |  |
| 18-39 | 307 | 329 | 354 | 387 | 427 | 477 |
| 40-49 | 783 | 877 | 927 | 973 | 1,038 | 1,134 |
| 50-59 | 2,243 | 2,458 | 2,837 | 3,183 | 3,366 | 3,542 |
| 60-69 | 4,301 | 4,664 | 4,988 | 5,490 | 6,361 | 7,156 |
| 70-79 | 4,711 | 6,293 | 8,170 | 8,940 | 9,598 | 10,614 |
| 80-89 | 4,624 | 5,429 | 6,121 | 8,751 | 11,506 | 12,800 |
| 90+ | 1,445 | 1,817 | 2,219 | 2,881 | 3,457 | 5,582 |

## Calculation based on INSR data and Faran and Klinger 2018

Table S12: forecast of the annual number of acute cerebrovascular events in the adult population (18+) in Israel, high population growth scenario 2015-2040, assuming decreasing annual incidence rates at a rate of 2% every 5 years

|  | 2015 | 2020 | 2025 | 2030 | 2035 | 2040 |
| --- | --- | --- | --- | --- | --- | --- |
| Total | 18,415 | 21,430 | 24,603 | 28,806 | 32,978 | 37,336 |
| by gender and ethnicity |  |  |  |  |  |  |
| Arab men | 1,571 | 1,959 | 2,448 | 2,991 | 3,616 | 4,290 |
| Arab women | 1,184 | 1,463 | 1,841 | 2,267 | 2,795 | 3,379 |
| Jewish and other men | 8,341 | 9,643 | 10,954 | 12,604 | 14,172 | 15,747 |
| Jewish and other women | 7,318 | 8,365 | 9,360 | 10,943 | 12,396 | 13,920 |
| by age group |  |  |  |  |  |  |
| 18-39 | 307 | 322 | 340 | 365 | 394 | 431 |
| 40-49 | 783 | 860 | 890 | 916 | 958 | 1,025 |
| 50-59 | 2,243 | 2,409 | 2,725 | 2,996 | 3,105 | 3,202 |
| 60-69 | 4,301 | 4,571 | 4,791 | 5,167 | 5,867 | 6,468 |
| 70-79 | 4,711 | 6,167 | 7,847 | 8,414 | 8,853 | 9,594 |
| 80-89 | 4,624 | 5,320 | 5,879 | 8,236 | 10,613 | 11,571 |
| 90+ | 1,445 | 1,781 | 2,131 | 2,712 | 3,189 | 5,046 |

## Calculation based on INSR data and Faran and Klinger 2018

Table S13: forecast of the annual number of acute cerebrovascular events in the adult population (18+) in Israel, high population growth scenario 2015-2040, assuming decreasing annual incidence rates at a rate of 7.25% every 5 years

|  | 2015 | 2020 | 2025 | 2030 | 2035 | 2040 |
| --- | --- | --- | --- | --- | --- | --- |
| Total | 18,415 | 20,282 | 22,037 | 24,420 | 26,459 | 28,351 |
| by gender and ethnicity |  |  |  |  |  |  |
| Arab men | 1,571 | 1,854 | 2,192 | 2,535 | 2,901 | 3,257 |
| Arab women | 1,184 | 1,385 | 1,649 | 1,922 | 2,242 | 2,565 |
| Jewish and other men | 8,341 | 9,126 | 9,812 | 10,685 | 11,370 | 11,958 |
| Jewish and other women | 7,318 | 7,917 | 8,384 | 9,277 | 9,946 | 10,570 |
| by age group |  |  |  |  |  |  |
| 18-39 | 307 | 305 | 305 | 309 | 316 | 327 |
| 40-49 | 783 | 814 | 797 | 777 | 768 | 778 |
| 50-59 | 2,243 | 2,280 | 2,441 | 2,540 | 2,491 | 2,431 |
| 60-69 | 4,301 | 4,326 | 4,291 | 4,381 | 4,707 | 4,912 |
| 70-79 | 4,711 | 5,837 | 7,028 | 7,133 | 7,103 | 7,285 |
| 80-89 | 4,624 | 5,035 | 5,266 | 6,982 | 8,515 | 8,786 |
| 90+ | 1,445 | 1,686 | 1,909 | 2,299 | 2,559 | 3,831 |

## Calculation based on INSR data and Faran and Klinger 2018

Figure S1: Forecast of the annual number of acute cerebrovascular events within the adult population (18+), for alternative population scenarios 2015-2040, under various assumption about stroke incidence rate

1. Forecast by type of event based on CBS intermediate population growth scenario

Table S14: Subgroup-specific mean annual incidence rate of acute cerebrovascular events in Israel during 2014-2016, by gender, ethnicity, age group and type of event (number of cases per 1,000)

| Hemorrhagic stroke | | | | | |
| --- | --- | --- | --- | --- | --- |
|  | Jews and Others | | Arabs | | Total |
| Age group | Men | Women | Men | Women |  |
| 18-39 | 0.02 | 0.01 | 0.03 | 0.01 | 0.02 |
| 40-49 | 0.06 | 0.03 | 0.10 | 0.06 | 0.05 |
| 50-59 | 0.22 | 0.09 | 0.30 | 0.17 | 0.17 |
| 60-69 | 0.48 | 0.21 | 0.82 | 0.48 | 0.37 |
| 70-79 | 1.19 | 0.72 | 1.76 | 1.32 | 0.99 |
| 80-89 | 2.27 | 1.55 | 2.28 | 2.66 | 1.87 |
| 90+ | 2.70 | 1.96 | 2.50 | 1.39 | 2.21 |
| Total | 0.28 | 0.20 | 0.21 | 0.16 | 0.23 |
| Ischemic stroke | | | | | |
|  | Jews and Others | | Arabs | | Total |
| Age group | Men | Women | Men | Women |  |
| 18-39 | 0.05 | 0.05 | 0.08 | 0.06 | 0.06 |
| 40-49 | 0.51 | 0.27 | 0.97 | 0.45 | 0.45 |
| 50-59 | 2.23 | 0.98 | 4.00 | 2.05 | 1.82 |
| 60-69 | 5.11 | 2.25 | 9.32 | 4.95 | 3.94 |
| 70-79 | 9.53 | 6.22 | 15.36 | 12.02 | 8.26 |
| 80-89 | 16.07 | 14.82 | 19.95 | 21.18 | 15.63 |
| 90+ | 22.42 | 22.71 | 20.00 | 18.89 | 22.46 |
| Total | 2.41 | 1.94 | 2.02 | 1.48 | 2.09 |
| Transient ischemic attack | | | | | |
|  | Jews and Others | | Arabs | | Total |
| Age group | Men | Women | Men | Women |  |
| 18-39 | 0.03 | 0.05 | 0.06 | 0.06 | 0.05 |
| 40-49 | 0.30 | 0.23 | 0.58 | 0.35 | 0.30 |
| 50-59 | 0.92 | 0.60 | 1.65 | 1.07 | 0.85 |
| 60-69 | 2.14 | 1.38 | 3.35 | 2.10 | 1.84 |
| 70-79 | 4.02 | 2.96 | 4.68 | 4.20 | 3.53 |
| 80-89 | 6.71 | 5.93 | 5.67 | 6.54 | 6.24 |
| 90+ | 8.20 | 7.19 | 2.08 | 4.72 | 7.38 |
| Total | 1.02 | 0.92 | 0.77 | 0.63 | 0.92 |

INSR data and CBS annual statistical reports

Table S15: forecast of the annual number of acute cerebrovascular events in the adult population (18+) in Israel, intermediate population growth scenario 2015-2040, assuming constant annual incidence rates, by event type, gender and ethnicity

|  | 2015 | 2020 | 2025 | 2030 | 2035 | 2040 |
| --- | --- | --- | --- | --- | --- | --- |
| Hemorrhagic stroke | | | | | | |
| Total | 1,315 | 1,554 | 1,803 | 2,136 | 2,469 | 2,809 |
| Arab men | 109 | 138 | 174 | 217 | 268 | 324 |
| Arab women | 82 | 103 | 132 | 165 | 208 | 257 |
| Jewish and other men | 639 | 753 | 865 | 1,012 | 1,149 | 1,283 |
| Jewish and other women | 484 | 560 | 632 | 742 | 843 | 944 |
| Ischemic stroke | | | | | | |
| Total | 11,879 | 13,967 | 16,156 | 19,081 | 22,032 | 25,176 |
| Arab men | 1,059 | 1,344 | 1,706 | 2,118 | 2,598 | 3,121 |
| Arab women | 774 | 968 | 1,236 | 1,546 | 1,938 | 2,373 |
| Jewish and other men | 5,410 | 6,311 | 7,213 | 8,338 | 9,424 | 10,541 |
| Jewish and other women | 4,635 | 5,344 | 6,000 | 7,079 | 8,072 | 9,141 |
| Transient ischemic attack | | | | | | |
| Total | 5,221 | 6,095 | 6,999 | 8,163 | 9,333 | 10,524 |
| Arab men | 402 | 505 | 630 | 769 | 927 | 1,100 |
| Arab women | 328 | 407 | 510 | 626 | 767 | 924 |
| Jewish and other men | 2,292 | 2,671 | 3,048 | 3,514 | 3,964 | 4,411 |
| Jewish and other women | 2,199 | 2,512 | 2,812 | 3,254 | 3,676 | 4,089 |

Calculation based on INSR data and Faran and Klinger 2018

Table S16: forecast of the annual number of acute cerebrovascular events in the adult population (18+) in Israel, intermediate population growth scenario 2015-2040, assuming constant annual incidence rates, by event type and age group

|  | 2015 | 2020 | 2025 | 2030 | 2035 | 2040 |
| --- | --- | --- | --- | --- | --- | --- |
| Hemorrhagic stroke | | | | | | |
| Total | 1,315 | 1,554 | 1,803 | 2,136 | 2,469 | 2,809 |
| 18-39 | 40 | 43 | 46 | 51 | 55 | 61 |
| 40-49 | 53 | 59 | 63 | 66 | 70 | 76 |
| 50-59 | 133 | 145 | 167 | 187 | 198 | 208 |
| 60-69 | 260 | 281 | 300 | 330 | 381 | 428 |
| 70-79 | 364 | 483 | 620 | 674 | 720 | 794 |
| 80-89 | 365 | 422 | 466 | 655 | 845 | 930 |
| 90+ | 100 | 121 | 140 | 173 | 200 | 313 |
| Ischemic stroke | | | | | | |
| Total | 11,879 | 13,967 | 16,156 | 19,081 | 22,032 | 25,176 |
| 18-39 | 145 | 155 | 167 | 183 | 201 | 220 |
| 40-49 | 436 | 489 | 516 | 542 | 579 | 632 |
| 50-59 | 1,436 | 1,575 | 1,816 | 2,034 | 2,148 | 2,261 |
| 60-69 | 2,757 | 2,989 | 3,199 | 3,521 | 4,068 | 4,566 |
| 70-79 | 3,047 | 4,032 | 5,182 | 5,644 | 6,055 | 6,684 |
| 80-89 | 3,044 | 3,504 | 3,859 | 5,411 | 6,977 | 7,677 |
| 90+ | 1,013 | 1,224 | 1,416 | 1,746 | 2,004 | 3,135 |
| Transient ischemic attack | | | | | | |
| Total | 5,221 | 6,095 | 6,999 | 8,163 | 9,333 | 10,524 |
| 18-39 | 122 | 130 | 140 | 153 | 168 | 184 |
| 40-49 | 293 | 329 | 347 | 364 | 388 | 423 |
| 50-59 | 673 | 735 | 847 | 949 | 1,002 | 1,054 |
| 60-69 | 1,285 | 1,379 | 1,457 | 1,589 | 1,834 | 2,058 |
| 70-79 | 1,300 | 1,725 | 2,215 | 2,385 | 2,521 | 2,757 |
| 80-89 | 1,215 | 1,394 | 1,526 | 2,148 | 2,765 | 3,016 |
| 90+ | 333 | 403 | 466 | 574 | 656 | 1,031 |

Calculation based on INSR data and Faran and Klinger 2018

Table S17: forecast of the annual number of acute cerebrovascular events in the adult population (18+) in Israel, intermediate population growth scenario 2015-2040, assuming decreasing annual incidence rates at a rate of 2% every 5 years, by event type, gender and ethnicity

|  | 2015 | 2020 | 2025 | 2030 | 2035 | 2040 |
| --- | --- | --- | --- | --- | --- | --- |
| Hemorrhagic stroke | | | | | | |
| Total | 1,315 | 1,523 | 1,732 | 2,010 | 2,278 | 2,539 |
| Arab men | 109 | 135 | 167 | 204 | 248 | 293 |
| Arab women | 82 | 101 | 127 | 155 | 192 | 232 |
| Jewish and other men | 639 | 738 | 831 | 952 | 1,060 | 1,160 |
| Jewish and other women | 484 | 549 | 607 | 699 | 778 | 854 |
| Ischemic stroke | | | | | | |
| Total | 11,879 | 13,688 | 15,516 | 17,959 | 20,322 | 22,757 |
| Arab men | 1,059 | 1,317 | 1,639 | 1,993 | 2,396 | 2,821 |
| Arab women | 774 | 949 | 1,187 | 1,455 | 1,787 | 2,145 |
| Jewish and other men | 5,410 | 6,185 | 6,927 | 7,848 | 8,693 | 9,528 |
| Jewish and other women | 4,635 | 5,237 | 5,763 | 6,663 | 7,446 | 8,263 |
| Transient ischemic attack | | | | | | |
| Total | 5,221 | 5,973 | 6,722 | 7,683 | 8,609 | 9,513 |
| Arab men | 402 | 495 | 605 | 724 | 855 | 994 |
| Arab women | 328 | 399 | 490 | 589 | 708 | 835 |
| Jewish and other men | 2,292 | 2,617 | 2,927 | 3,307 | 3,656 | 3,987 |
| Jewish and other women | 2,199 | 2,462 | 2,700 | 3,063 | 3,390 | 3,696 |

Calculation based on INSR data and Faran and Klinger 2018

Table S18: forecast of the annual number of acute cerebrovascular events in the adult population (18+) in Israel, intermediate population growth scenario 2015-2040, assuming decreasing annual incidence rates at a rate of 2% every 5 years, by event type and age group

|  | 2015 | 2020 | 2025 | 2030 | 2035 | 2040 |
| --- | --- | --- | --- | --- | --- | --- |
| Hemorrhagic stroke | | | | | | |
| Total | 1,315 | 1,523 | 1,732 | 2,010 | 2,278 | 2,539 |
| 18-39 | 40 | 42 | 44 | 48 | 51 | 55 |
| 40-49 | 53 | 58 | 60 | 62 | 65 | 69 |
| 50-59 | 133 | 142 | 161 | 176 | 183 | 188 |
| 60-69 | 260 | 275 | 288 | 310 | 351 | 387 |
| 70-79 | 364 | 473 | 596 | 634 | 664 | 717 |
| 80-89 | 365 | 413 | 448 | 617 | 779 | 840 |
| 90+ | 100 | 118 | 135 | 163 | 184 | 283 |
| Ischemic stroke | | | | | | |
| Total | 11,879 | 13,688 | 15,516 | 17,959 | 20,322 | 22,757 |
| 18-39 | 145 | 152 | 161 | 172 | 185 | 199 |
| 40-49 | 436 | 479 | 495 | 510 | 534 | 571 |
| 50-59 | 1,436 | 1,543 | 1,744 | 1,914 | 1,981 | 2,044 |
| 60-69 | 2,757 | 2,929 | 3,072 | 3,314 | 3,752 | 4,127 |
| 70-79 | 3,047 | 3,951 | 4,977 | 5,313 | 5,585 | 6,042 |
| 80-89 | 3,044 | 3,434 | 3,707 | 5,092 | 6,436 | 6,940 |
| 90+ | 1,013 | 1,199 | 1,360 | 1,643 | 1,848 | 2,834 |
| Transient ischemic attack | | | | | | |
| Total | 5,221 | 5,973 | 6,722 | 7,683 | 8,609 | 9,513 |
| 18-39 | 122 | 128 | 135 | 144 | 155 | 166 |
| 40-49 | 293 | 322 | 333 | 343 | 358 | 382 |
| 50-59 | 673 | 721 | 814 | 893 | 925 | 953 |
| 60-69 | 1,285 | 1,351 | 1,399 | 1,496 | 1,691 | 1,860 |
| 70-79 | 1,300 | 1,691 | 2,128 | 2,245 | 2,325 | 2,492 |
| 80-89 | 1,215 | 1,366 | 1,466 | 2,022 | 2,550 | 2,727 |
| 90+ | 333 | 395 | 448 | 540 | 605 | 932 |

Calculation based on INSR data and Faran and Klinger 2018

Table S19: forecast of the annual number of acute cerebrovascular events in the adult population (18+) in Israel, intermediate population growth scenario 2015-2040, assuming decreasing annual incidence rates at a rate of 7.25% every 5 years, by event type, gender and ethnicity

|  | 2015 | 2020 | 2025 | 2030 | 2035 | 2040 |
| --- | --- | --- | --- | --- | --- | --- |
| Hemorrhagic stroke | | | | | | |
| Total | 1,315 | 1,441 | 1,551 | 1,704 | 1,827 | 1,928 |
| Arab men | 109 | 128 | 150 | 173 | 199 | 223 |
| Arab women | 82 | 96 | 113 | 132 | 154 | 176 |
| Jewish and other men | 639 | 698 | 744 | 807 | 850 | 881 |
| Jewish and other women | 484 | 520 | 544 | 592 | 624 | 648 |
| Ischemic stroke | | | | | | |
| Total | 11,879 | 12,955 | 13,898 | 15,224 | 16,305 | 17,281 |
| Arab men | 1,059 | 1,246 | 1,468 | 1,690 | 1,922 | 2,142 |
| Arab women | 774 | 898 | 1,064 | 1,233 | 1,434 | 1,628 |
| Jewish and other men | 5,410 | 5,854 | 6,205 | 6,653 | 6,974 | 7,235 |
| Jewish and other women | 4,635 | 4,957 | 5,162 | 5,648 | 5,974 | 6,275 |
| Transient ischemic attack | | | | | | |
| Total | 5,221 | 5,653 | 6,021 | 6,513 | 6,907 | 7,223 |
| Arab men | 402 | 468 | 542 | 613 | 686 | 755 |
| Arab women | 328 | 378 | 439 | 500 | 568 | 634 |
| Jewish and other men | 2,292 | 2,477 | 2,622 | 2,804 | 2,933 | 3,028 |
| Jewish and other women | 2,199 | 2,330 | 2,419 | 2,597 | 2,720 | 2,807 |

Calculation based on INSR data and Faran and Klinger 2018

Table S20: forecast of the annual number of acute cerebrovascular events in the adult population (18+) in Israel, intermediate population growth scenario 2015-2040, assuming decreasing annual incidence rates at a rate of 7.25% every 5 years, by event type and age group

|  | 2015 | 2020 | 2025 | 2030 | 2035 | 2040 |
| --- | --- | --- | --- | --- | --- | --- |
| Hemorrhagic stroke | | | | | | |
| Total | 1,315 | 1,441 | 1,551 | 1,704 | 1,827 | 1,928 |
| 18-39 | 40 | 40 | 40 | 40 | 41 | 40 |
| 40-49 | 53 | 55 | 54 | 53 | 52 | 53 |
| 50-59 | 133 | 135 | 144 | 150 | 147 | 133 |
| 60-69 | 260 | 261 | 258 | 263 | 282 | 260 |
| 70-79 | 364 | 448 | 534 | 538 | 533 | 364 |
| 80-89 | 365 | 391 | 401 | 523 | 625 | 365 |
| 90+ | 100 | 112 | 121 | 138 | 148 | 100 |
| Ischemic stroke | | | | | | |
| Total | 11,879 | 12,955 | 13,898 | 15,224 | 16,305 | 17,281 |
| 18-39 | 145 | 144 | 144 | 146 | 149 | 151 |
| 40-49 | 436 | 453 | 444 | 432 | 428 | 434 |
| 50-59 | 1,436 | 1,461 | 1,562 | 1,623 | 1,590 | 1,552 |
| 60-69 | 2,757 | 2,772 | 2,752 | 2,809 | 3,011 | 3,134 |
| 70-79 | 3,047 | 3,739 | 4,458 | 4,504 | 4,481 | 4,588 |
| 80-89 | 3,044 | 3,250 | 3,320 | 4,317 | 5,164 | 5,270 |
| 90+ | 1,013 | 1,135 | 1,218 | 1,393 | 1,483 | 2,152 |
| Transient ischemic attack | | | | | | |
| Total | 5,221 | 5,653 | 6,021 | 6,513 | 6,907 | 7,223 |
| 18-39 | 122 | 121 | 121 | 122 | 125 | 126 |
| 40-49 | 293 | 305 | 298 | 290 | 287 | 290 |
| 50-59 | 673 | 682 | 729 | 757 | 742 | 723 |
| 60-69 | 1,285 | 1,279 | 1,253 | 1,268 | 1,357 | 1,412 |
| 70-79 | 1,300 | 1,600 | 1,906 | 1,903 | 1,866 | 1,893 |
| 80-89 | 1,215 | 1,293 | 1,313 | 1,714 | 2,046 | 2,070 |
| 90+ | 333 | 374 | 401 | 458 | 485 | 708 |

Calculation based on INSR data and Faran and Klinger 2018

Figure S2: Forecast of the annual number of acute cerebrovascular events within the adult population, 2015-2040, intermediate population growth scenario, under various assumption about stroke incidence rate, by type of event
